# Editorial: Immune-boosting effects of dietary bioactive polysaccharides

**DOI:** 10.3389/fnut.2022.1102641

**Published:** 2022-12-02

**Authors:** Bin Du, Baojun Xu

**Affiliations:** ^1^Hebei Key Laboratory of Natural Products Activity Components and Function, Hebei Normal University of Science and Technology, Qinhuangdao, China; ^2^Food Science and Technology Program, Department of Life Sciences, BNU–HKBU United International College, Zhuhai, China

**Keywords:** immunomodulation, polysaccharides, cell signaling, molecular pathways, cellular mechanisms

In recent year, polysaccharides currently represent a hot research field. Dietary bioactive polysaccharides have attracted more attention due to its non-harmful and non-toxic properties (1, 2). Abundant studies have identified valuable biological activities of dietary bioactive polysaccharides, especially immunomodulating activity (3, 4). Previous studies have shown that the mechanisms involved in immunomodulating effects are due to the modulation of innate immunity and macrophage function (5). However, the underlying cellular signaling and molecular mechanisms of their immune-boosting activity are not clear.

This Research Topic is aimed at collecting and summarizing the immune-boosting effects of dietary bioactive polysaccharides (such as inulin, dietary gum, fucoidan, glucan, glucomannan, heteropolysaccharides, etc.) from different natural sources (such as fruits and vegetables, cereal grains, edible mushrooms, sea foods, medicinal plants) in immune cells, animal study and clinical study as well.

In this special e-collection there are nine papers covering the above-mentioned aspects. Roselli et al. investigated the anti-inflammatory activity of galactooligosaccharide in an *in vitro* model of ulcerative colitis (UC)-like inflamed intestinal cells. The findings indicated that Bimuno GOS at different concentrations, while not affecting cell monolayer permeability, was shown to counteract UC-like intestinal inflammatory responses and damages induced by DSS. Indeed, Bimuno GOS was able to counteract the detrimental effects of DSS on cell permeability, determined by transepithelial electrical resistance, phenol red apparent permeability, and tight- and adherent junction protein distribution. Furthermore, Bimuno GOS inhibited the DSS-induced NF-kB nuclear translocation and pro-inflammatory cytokine secretion. Further analyses showed that Bimuno GOS was able to revert the expression levels of most of the proteins involved in the NF-κB cascade to control levels (Roselli et al.).

*Lycium barbarum* polysaccharides have been widely explored for their potential health properties. Zhang et al. tested the mechanisms of *L. barbarum* residue (RW) and fermented *L. barbarum* residue (RFW) on meat quality and immunity of sheep. Fifty-four Tan sheep were randomly divided into control, RFW or RW treatments. Data showed that RFW and RW increased the carcass weight, fat content, ash content and reduced the cooking loss of lamb. RFW performed more significant effects on activating immune-related genes than those of RW. The expression of chemokines and immune-related pathways, such as signaling pathways of interleukin-17 signaling pathway and NOD-like receptor signaling pathway, were elevated in sheep fed RFW. RW increased the diversity in rumen metabolites, especially compositions of lipids, organic acids and organ heterocyclic compounds (Zhang et al.).

Park et al. assessed the immune-enhancing effect of co-treatment with *Kalopanax pictus Nakai* Bark and *Nelumbo nucifera* Gaertner leaf extract (KPNN) in a cyclophosphamide (Cy)-induced immunosuppressed rat model. KPNN significantly increased phospho-NF-κB and phospho-ERK protein levels and cell viability in macrophages. KPNN significantly increased the NK cell activity in splenocytes compared to that in the control. Cy treatment decreased tumor necrosis factor (TNF)-α, interleukin (IL)-6, and interferon-γ production. In the Cy-induced immunosuppression rat model, KPNN-treated rats had significantly higher body weights and tissue weights than the Cy-treated rats. Additionally, KPNN treatment restored the immune-related factors, such as total leukocyte, lymphocyte, and intermediate cell contents, to their normal levels in the blood. The blood cytokines (TNF-α and IL-6) were increased, and spleen tissue damage was significantly alleviated (Park et al.).

In this Research Topic, there are two papers covering the fucoidan research. Fucoidan is a type of polysaccharide rich in sulfuric acid groups and is mainly found in brown algae. In this study, the effect of sterile fucoidan on the T-cell response and the subsequent modulation of osteogenesis is investigated. The physicochemical features of fucoidan treated by high-temperature autoclave sterilization are characterized by UV–visible spectroscopy, X-ray diffraction, Fourier transform infrared and nuclear magnetic resonance analysis. It is demonstrated that high-temperature autoclave treatment resulted in fucoidan depolymerization, with no change in its key bioactive groups. Further, sterile fucoidan promotes T cells proliferation and the proportion of differentiated T cells decreases with increasing concentration of fucoidan. In addition, the supernatant of T cells co-cultured with fucoidan greatly suppresses the osteogenic differentiation of MC3T3-E1 by downregulating the formation of alkaline phosphatase and calcium nodule compared with fucoidan (Huang et al.).

In another study, the effects of *Laminaria japonica* fucoidan (LF) on immune regulation and intestinal microflora in cyclophosphamide (CTX)-treated mice were investigated in this work. Results indicated that LF significantly enhanced the spleen and thymus indices, promoted spleen lymphocyte and peritoneal macrophages proliferation, and increased the immune-related cytokines production in serum. Moreover, LF could regulate intestinal flora composition, increasing the abundance of *Lactobacillaceae* and *Alistipes*, and inhibiting *Erysipelotrichia, Turicibacter, Romboutsia, Peptostreptococcaceae*, and *Faecalibaculum* (Tang et al.).

Yang et al. investigated the structural characterization and immunological activity *in vitro* and *in vivo* of a polysaccharide from the rhizome of *Menispermum dauricum*. A new polysaccharide named MDP was isolated from the rhizome of *M. dauricum* by hot water extraction, ethanol precipitation, anion-exchange, and gel-filtration chromatography. MDP was homogeneous and had a molecular weight of 6.16 × 10^3^ Da, and it was an α-D-glucan containing a (1 → 6)-linked backbone, with a glucosyl residue at the C-3 position along the main chain. MDP exhibited immunological activity *in vitro*, which could significantly promote the proliferation and phagocytosis of RAW264.7 cells and the release of TNF-α and IL-6 factors. MDP also could significantly increase the thymus and spleen indices, enhance the macrophage function, increase the level of cytokine (IL-6 and TNF-α) and immunoglobulin IgM in the serum and regulate T lymphocyte subsets (Yang et al.).

Both edible and medicinal mushrooms possess strong therapeutic and biological activities (6). There are two papers covering the immune-boosting effects of mushroom polysaccharides in this special e-collection. *Flammulina velutipes* polysaccharides could improve gut health through gut microbiota and metabolism regulation. Data from Liang et al. showed that compared with the model group, *F. velutipes* polysaccharide could increase thymus and spleen indices and improve thymus tissue structure in mice; IL-2 and IL-4 contents were significantly increased and IL-6 and TNF-α contents were significantly decreased; serum acid phosphatase (ACP), lactate dehydrogenase (LDH) and total antioxidant capacity (T-AOC) activities were increased (*P* < 0.05); in the liver, superoxide dismutase (SOD) and catalase (CAT) activities were increased (*P* < 0.001), while malondialdehyde (MDA) content was decreased (*P* < 0.001). Proteomics discovered that *F. velutipes* polysaccharides may exert immune modulatory effects by participating in signaling pathways such as immune diseases, transport and catabolism, phagosomes and influenza A, regulating the immune-related proteins Transferrin receptor protein 1 (TFRC) and Radical S-adenosyl methionine domain-containing protein 2 (RSAD2), etc. Gut microbial studies showed that *F. velutipes* polysaccharides could increase the abundance of intestinal flora and improve the flora structure (Liang et al.).

Furthermore, to investigate the effect of *F. velutipes* polysaccharides (FVPs) on mice intestinal inflammation, FVPs were extracted from *F. velutipes* (FV) using a solid anaerobic fermentation technique. The antioxidant and anti-inflammatory capacities of FVP and fermented FVP (FFVP) induced by lipopolysaccharide (LPS) were investigated *in vitro* and *in vivo*. The results showed that the yield of FFVP (9.44%) was higher than that of FVP (8.65%), but the molecular weight (MW) of FFVP (15,702 Da) was lower than that of FVP (15,961 Da). The antioxidant and anti-inflammatory capacities of FFVP were higher than that of FVP in preventing mice diarrhea, enhancing antioxidant capacities, and reducing the secretion and mRNA expression of interleukin-1β (IL-1β), IL-6, IL-18, and tumor necrosis factor-α (TNF-α). The anti-inflammatory mechanisms of FVP and FFVP were analyzed by inhibiting the activation of the NLRP3 signaling pathway using an LPS-induced mice model (Ma et al.).

Li et al. employed RNA-sequencing (RNA-seq) to determine the level and function of differentially expressed genes (DEGs) and further explore the mechanism of the HRP anti-inflammatory and immune process. The differential expression analysis indicated that 3622, 1216, and 2100 DEGs in the IPEC-J2 cells were identified in C vs. L, L vs. H6-L, and C vs. H6-L, respectively. The Kyoto Encyclopedia of Genes and Genomes (KEGG) enrichment analysis found six identified pathways related to the immune system. Additionally, the authors used the Science, Technology, Engineering, and Math (STEM) program to categorize the 3,134 DEGs that were differentially expressed in H2-L, H4-L and H6-L into eight possible expression profiles, in which 612 were clustered into two profiles. The accuracy and consistency of RNA-seq data were validated by the results of qRT-PCR of the nuclear factor of kappa light polypeptide gene enhancer in B-cells 2 (NFKB2), MAP kinase interacting serine/threonine kinase 2 (MKNK2), mitogen-activated protein kinase 1 (MAP2K1), mitogen-activated protein kinase kinase kinase 8 (MAP3K8), Ras-related protein R-Ras (RRAS), TNF receptor-associated factor 1 (TRAF1), NF-kappa-B inhibitor alpha (NFKBIA), interleukin 8 (IL8), tumor necrosis factor, alpha-induced protein 3 (TNFAIP3), and transforming growth factor beta-1 (TGFB1). Transcriptome sequencing also indicated that HRP reduced the expression levels of related DEGs and inhibited the activation of the mitogen-activated protein kinase (MAPK)/nuclear factor kappa-B (NF-κB) signaling pathway (Li et al.).

In summary, the results of the above-mentioned studies will fill the gap between the knowledge on significant therapeutic immune-boosting activities of dietary polysaccharides and their underlying cellular signaling and molecular mechanisms. It also aims to accumulate new knowledge and methods for discovery and development of novel therapeutic agents and adjuvants that exhibit beneficial immune-boosting properties.

## Author contributions

BD wrote the introduction and the conclusion. BX wrote the central part with comments to the cited papers and references. Both authors contributed to the article and approved the submitted version.

## Conflict of interest

The authors declare that the research was conducted in the absence of any commercial or financial relationships that could be construed as a potential conflict of interest.

## Publisher's note

All claims expressed in this article are solely those of the authors and do not necessarily represent those of their affiliated organizations, or those of the publisher, the editors and the reviewers. Any product that may be evaluated in this article, or claim that may be made by its manufacturer, is not guaranteed or endorsed by the publisher.
